# Metabolic consequences of long-term rapamycin exposure on common marmoset monkeys (*Callithrix jacchus*)

**DOI:** 10.18632/aging.100843

**Published:** 2015-11-13

**Authors:** Corinna Ross, Adam Salmon, Randy Strong, Elizabeth Fernandez, Marty Javors, Arlan Richardson, Suzette Tardif

**Affiliations:** ^1^ Department of Arts & Sciences, Texas A&M University San Antonio, San Antonio, TX 78224, USA; ^2^ Barshop Institute for Longevity & Aging Studies, University of Texas Health Science Center San Antonio, San Antonio, TX 78224, USA; ^3^ Geriatric Research, Education & Clinical Center, South Texas Veteran's Health Care System, San Antonio, TX 78224, USA; ^4^ University of Oklahoma Health Sciences Center and the Oklahoma City VA Medical Center, Oklahoma City, OK 73104, USA; ^5^ Southwest National Primate Research Center, Texas Biomedical Research Institute, San Antonio, TX 78224, USA

**Keywords:** nonhuman primate, antiaging, longevity, healthspan, animal models, sirolimus

## Abstract

Rapamycin has been shown to extend lifespan in rodent models, but the effects on metabolic health and function have been widely debated in both clinical and translational trials. Prior to rapamycin being used as a treatment to extend both lifespan and healthspan in the human population, it is vital to assess the side effects of the treatment on metabolic pathways in animal model systems, including a closely related non-human primate model. In this study, we found that long-term treatment of marmoset monkeys with orally-administered encapsulated rapamycin resulted in no overall effects on body weight and only a small decrease in fat mass over the first few months of treatment. Rapamycin treated subjects showed no overall changes in daily activity counts, blood lipids, or significant changes in glucose metabolism including oral glucose tolerance. Adipose tissue displayed no differences in gene expression of metabolic markers following treatment, while liver tissue exhibited suppressed G6Pase activity with increased PCK and GPI activity. Overall, the marmosets revealed only minor metabolic consequences of chronic treatment with rapamycin and this adds to the growing body of literature that suggests that chronic and/or intermittent rapamycin treatment results in improved health span and metabolic functioning. The marmosets offer an interesting alternative animal model for future intervention testing and translational modeling.

## INTRODUCTION

Rapamycin has been found by multiple laboratories to extend mouse lifespan even when mice began receiving rapamycin relatively late in life at 20 months of age, or roughly the equivalent of 55 human years [1]. In addition, rapamycin has been shown to delay the onset of several age-related diseases, including Alzheimer's disease, cardiovascular disease, and cancer in mouse models of these pathologies [2-5]. These findings have led to significant interest in the potential effects of rapamycin as an anti-aging intervention in humans particularly because rapamycin is already approved for use in cancer therapy and as an adjunct immuno-suppressive agent for transplant patients.

However, clinical administration of rapamycin has the potential for several side-effects include hyperlipidemia and hyperglycemia [6], raising concerns as to the potential negative impacts of rapamycin exposure in aged human populations that may have a high underlying prevalence of obesity- and age-induced insulin resistance and hyperlipidemia. However, results from clinical populations suggests that some side effects, such as hypertriglyceridemia, may improve over time and that others may be dose-dependent [6]. The nature of the human data accumulated thus far paints a far from complete picture of why and how rapamycin might affect metabolism and lipid trafficking. Moreover, these clinical studies have focused on patients with pre-existing conditions and have largely utilized combination therapies with other immunosupressants or steroids that can cause metabolic dysfunctions of their own [7]. There have, however, been no long-term studies of the effects of monothereapy with rapamycin or its analogs in populations of otherwise healthy humans. Thus, it is not clear whether the previously reported metabolic risks of rapamycin and its analogs are due to this drug directly or to other confounding factors.

In rodent models, monotherapy with rapamycin has largely, though not equivocally, been associated with impairment of glucose metabolism as measured in glucose tolerance tests. A notable exception is a study by Fang et al. suggesting that chronic treatment with rapamycin has a biphasic effect on glucose metabolism with short-term rapamycin treatment being detrimental to glucose metabolism whereas long-term (20 wk) treatment with rapamycin may promote an insulin-sensitive state in mice with a transition state in between [8]. Further examination has revealed that long term treatment of rapamycin leads to a metabolic switch resulting in enhanced insulin signaling and better triglyceride processing [9]. These rodent studies have largely been performed in animals maintained on a relatively standardized rodent chow, though there is evidence that rapamycin has similar effects on both lifespan and metabolic function in mice fed diets high in caloric content due to increased levels of sugar and/or fat [10,11]. Mice given intermittent treatment of rapamycin and placed on a high fat diet have no gross changes in metabolic markers, and rapa appears to prevent weight gain [12]. Combining the intervention therapies of rapamycin and resveratrol treatment in mice was found to prevent insulin resistance in mice being fed a high fat diet and suggests that combination therapy may be beneficial in a high fat environment [13]. Human glucose metabolism is regulated by a complex interaction of genetics and environment (including diet) that cannot be fully recapitulated in laboratory rodents [14]. Even the timing of or causes of eating/overeating differ between rodent models and humans, further complicating this issue [15, 16]. At the molecular level, there are significant discrepancies between rodents and humans in alterations of gene regulation in metabolic dysfunction suggesting there is little overlap between the two models [17]. Lastly, many of the complications of metabolic dysfunction including nephropathy, neuropathy, and cardiac dysfunction cannot be successfully replicated in single genetic mouse models of metabolic dysfunction or in high fat-fed rodents [18]. Thus, a significant question remains whether the choice of diet (as well as sex of animals or background genetics) could potentially complicate the potential for translation [19-21]. An approach to address whether the effects (and potential side-effects like metabolic dysfunction) of rapamycin in otherwise healthy rodents may also be relevant to humans is to perform such experiments in other species that are predicted to have similar phenotypic metabolic regulation as humans. In other words, studies of rapamycin's effects in a species more closely related to humans can inform as to the generalizability of the rodent findings and issues likely to impede the general use of rapamycin as an anti-aging treatment in humans.

The common marmoset (*Callithrix jacchus*) is a small monkey with a relatively short lifespan. Both its small size and associated shorter lifespan make this species a valuable nonhuman primate model for the study of aging and chronic disease [22, 23]. Captive marmosets display many similarities to humans in terms of obesity and its sequelae. Spontaneous obesity has been described in multiple captive marmoset colonies that are socially housed and fed a relatively low fat, high fiber diet [22, 24-28]. Obesity in marmosets, defined in a fashion similar to that used in humans, is statistically associated with increased risk to metabolic dysfunction and cardiovascular disease [16]. In addition to displaying evidence of insulin resistance, marmosets at extremely high weights (over 500 grams) show higher age-specific mortality rates as adults when compared to animals of average weight [22]. From 2010-2011, we conducted a year-long study of daily dosing of a group of common marmosets with rapamycin. We previously reported that we were able to maintain circulating blood levels of rapamycin at 5.2 ng/mL by giving the animals a dose of eudragit encapsulated rapamycin in yogurt of 1mg/kg/day. Subjects demonstrated a decrease in mTORC1 after two weeks of treatment. There was no evidence of clinical anemia, mouth ulcers, lung fibrotic changes, significant changes in wound healing, or increased mortality [29]. This report describes a set of metabolic outcomes from this study.

## RESULTS

### Body Composition

**Figure 1:**
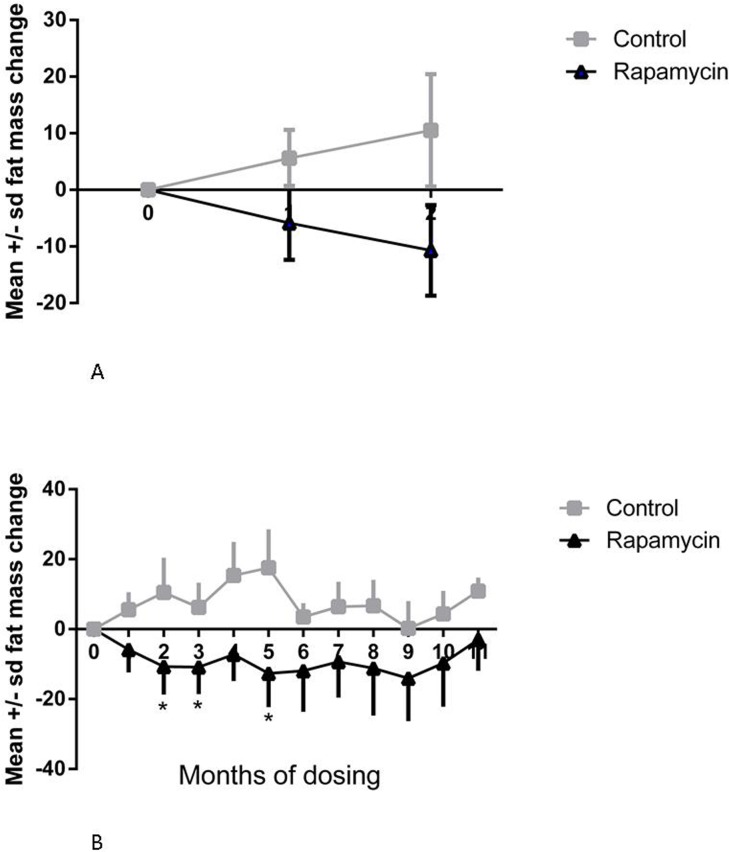
Change in fat mass (**A**) Change in fat mass at 1 and 2 months, post-dosing, from pre-dosing (month 0) measurement. Squares = control subjects; triangles = rapamycin subjects (mean ± SD); treatment × time interaction, p < 0.0097; difference in month 0 and month 2 mean for rapamycin treated subjects, p < 0.05, Sidak's multiple comparison test. (**B**) Change in fat mass from pre-dosing measurement for months 1-11 for rapamycin subjects. * treatment effect, F=5.385, p=0.018, Dunnett's multiple comparison test significant, p < 0.05, for month 0 versus months 2, 3, and 5.

Rapamycin treated subjects displayed a significant loss of body fat mass at two months post-dosing while control subjects displayed a statistically insignificant change in body fat mass, as illustrated in Fig. 1A (treatment × time interaction, p < 0.0097; difference in month 0 and month 2 mean for rapamycin treated subjects, p < 0.05, Sidak's multiple comparison test).

The rapamycin treated subjects had significantly reduced body fat mass in months 2, 3 and 5, after which their mean fat mass did not differ from the pre-dosing mean (treatment effect, F=5.385, p=0.018, Dunnett's multiple comparison test significant, p < 0.05, for month 0 versus months 2, 3, and 5), resulting in no difference between control and rapamycin treated subjects at the end of the study as illustrated in Fig 1B.

There were no significant differences between controls and rapamycin treated subjects and no effects of rapamycin treated subjects over time on body lean mass.

### Food intake and activity levels

**Figure 2:**
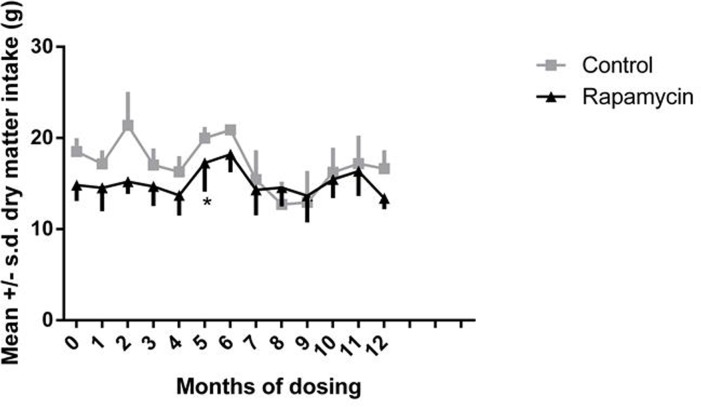
Food intake Daily dry matter intake for months 0-12, month 0 is a pre-dosing measurement. Squares = control subjects; triangles = rapamycin subjects (mean ± SD). * treatment effect, F=8.353, p=0.001, Dunnett's multiple comparison test significant, p < 0.05, for month 0 versus month 5.

There were no significant differences between controls and rapamycin treated subjects in food intake at two months post-dosing. In rapamycin-treated marmosets, there was a significant increase in food intake at month 5, over the pre-dosing food intake (treatment effect, F=8.353, p=0.001, Dunnett's multiple comparison test significant, p < 0.05, for month 0 versus month 5), as illustrated in Fig. 2.

**Figure 3:**
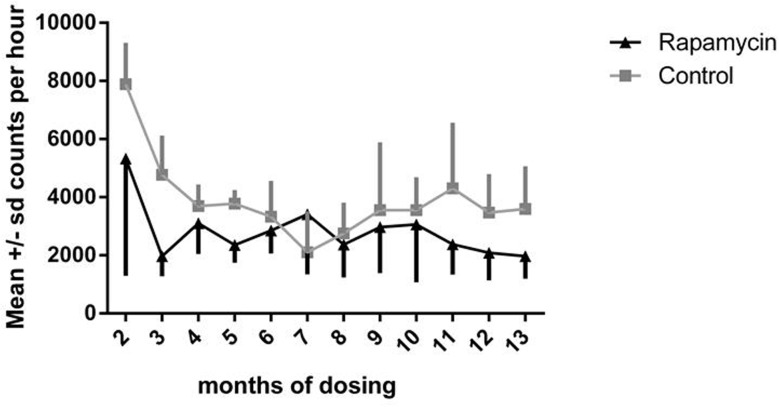
Daily activity Accelerometer counts per hour for months 2-13 of dosing. Squares = control subjects; triangles= rapamycin subjects (mean ± SD).

In both control and rapamycin-treated subjects activity scored as accelerometer counts per hour declined significantly after the second month of dosing, as illustrated in Fig. 3, then remained stable over the remainder of the study. There were no significant differences between controls and rapamycin treated subjects. Because both groups were affected, these data suggest that this decline was a result of habituation to wearing the harness holding the accelerometer.

### Lipid and glucose metabolic measures

There were no significant differences between controls and rapamycin-treated subjects in pre- versus post-dosing mean circulating triglyceride concentrations.

**Figure 4:**
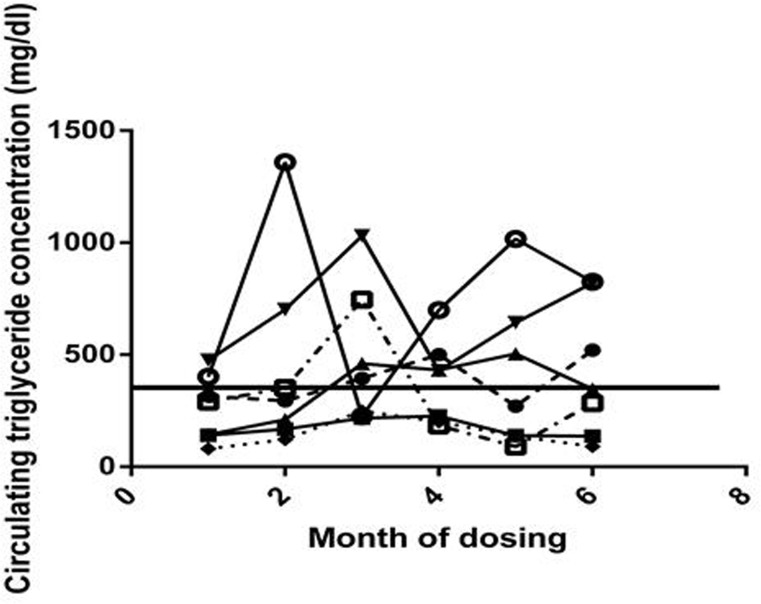
Circulating triglyceride Circulating triglyceride concentrations for each rapamycin subject for months 0-6, month 0 is a pre-dosing measurement. The solid horizontal line represents the previously established cut-off point for normal triglyceride concentrations in this species.

There was, however considerable inter-individual variation in both baseline triglyceride concentration and in change over time, as illustrated in Fig. 4. Two of the rapamycin treated subjects that were borderline hypertriglyceridemic (476 and 402 mg/dl, with > 400 mg/dl defined as hypertriglyceridemic, [24] before dosing, displayed dramatic increases in circulating triglyceride concentration at month 2 (702 and 1,359 mg/dl); however, their triglyceride concentrations then varied considerably over the next 7 months. There was no consistent hypertriglyceridemia caused by rapamycin among subjects who began with normal circulating triglyceride concentrations There were two subjects (one control and one rapamycin treated) that displayed severe hypertriglycerimedia before dosing (603 and 1,611 mg/dl respectively). They both remained hypertriglyceridemic through the study.

**Figure 5:**
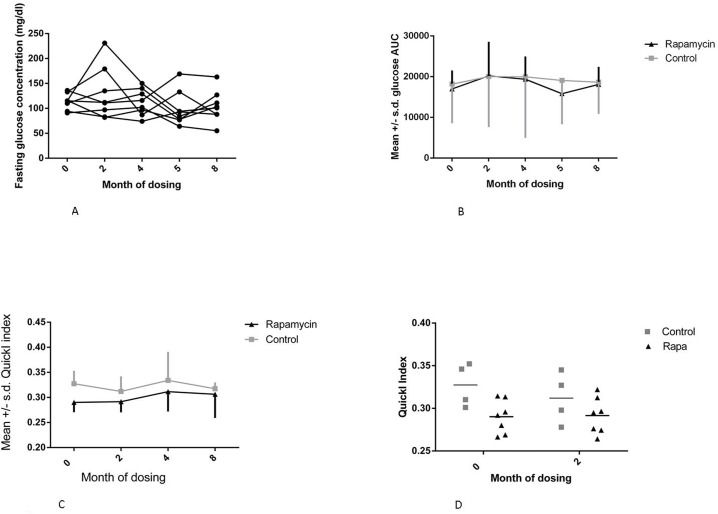
Metabolic measures (**A**) Fasting glucose concentration for months 0-8 for rapamycin subjects, month 0 is a pre-dosing measurement. (**B**) Glucose area under the curve (AUC) for months 0-8 for rapamycin subjects. (C) QuickI index for rapamycin subjects for months 0-8. (**D**) QuickI index - 1/[log(fasting insulin) + log(fasting glucose)] for months 0 and 2 of dosing, *(F=5.396, p = 0.0453, Sidak's multiple comparison test, p < 0.05 for month 0 control vs rapamycin treated). For all panels squares = control subjects; triangles = rapamycin subjects (mean ± SD).

Three measures of glucose metabolic function were assessed: fasting blood glucose, QuickI index, and AUC. There were no significant changes in fasting glucose concentrations as illustrated in Fig. 5A. The QuickI index, calculated as 1/[log(fasting insulin) + log(fasting glucose)], is the typical measure reported in nonhuman primate studies to provide an estimate of insulin sensitivity, with higher values indicating more insulin sensitivity. The area under the curve (AUC) for the glucose tolerance tests represents the relative glucose excursion caused by a consistent dose of glucose and is a measure of the efficiency with which the entire system can remove glucose from the circulation. As illustrated in Fig. 5D, the mean QuickI index for the control group was higher than that for the rapamycin treated group prior to treatment (F=5.396, p = 0.0453, Sidak's multiple comparison test, p < 0.05 for month 0 control vs rapamycin treated), suggesting that the animals that became the control group were, on average, more insulin sensitive than those in the group randomly selected to be treated with rapamycin. However, the rapamycin-treated group displayed a reduced QuickI measurement even prior to treatment that was not altered during these first two months of dosing as indicated by the lack of a significant interaction effect. We also found that QuickI did not differ among rapamycin-treated animals through 8 months of rapamycin treatment as illustrated in Fig. 5C.

There were no differences between control and rapamycin treated subjects and no interaction effect on the glucose AUC. There were also no significant differences in post-dosing average glucose AUC in the rapamycin treated subjects through 8 months of dosing, as illustrated in Fig. 5B. Together, these data suggest limited to no impairment of glucose metabolism in healthy marmosets treated with daily administration of rapamycin at doses sufficient to reduce mTOR signaling.

### Assessment of molecular effects

**Figure 6:**
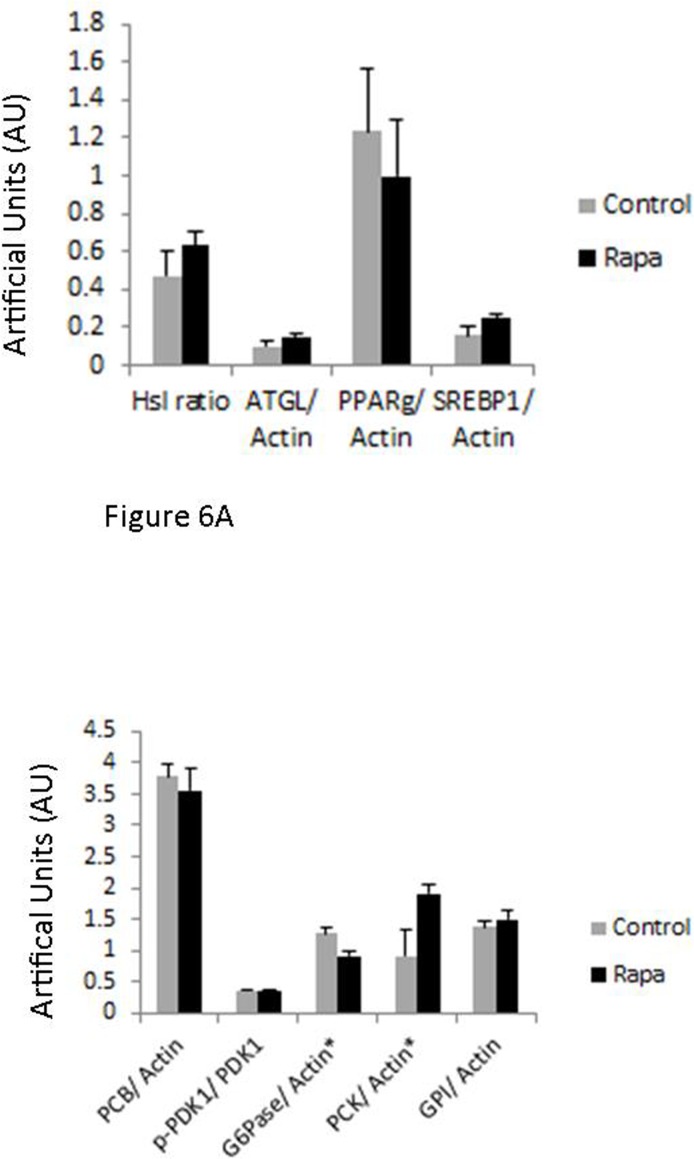
Immunoblot results Immunoblot results for the following: adipose triglyceride lipase (ATGL), pyruvate carboxylase (PCB), glucose-6-phosphatase α (G6Pase), glucose-6-phosphate isomerase (GPI), peroxisome proliferator-activated receptor γ (PPARγ), phospho-pyruvate dehydrogenase kinase (p-PDK1), pyruvate dehydrogenase kinase (PDK1), phosphoenol-pyruvate carboxykinase 1 (PCK1), sterol regulatory element-binding protein 1 (SREBP1) corrected by actin. A. Adipose tissue collected at sacrifice following 14 months of rapamycin (black) or control dosing (grey) (mean ± SE) B. Liver tissue collected at sacrifice following 14 months of rapamycin (black) or control dosing (grey) (mean ± SE) *indicates significance p<0.05.

The long-term administration of rapamycin in rodents has been associated with hyperglycemia caused in part by increased gluconeogenesis [27-29]. In the liver of rapamycin treated animals, we found significant upregulation of the expression of phosphoenolpyruvate carboxykinase (PCK1), indicative of altered gluconeo-genic capacity. Surprisingly, we found that glucose 6 phosphatase expression in rapamycin treated animals was significantly reduced (Fig. 6). Rapamycin did not alter the expression of other markers of gluconeogenesis. The lack of a consistent alteration in the expression of gluconeogenic effectors may explain why rapamycin-treated marmosets showed no significant change in fasting blood glucose levels.

Because rapamycin modulated fat content of marmosets in the early periods of treatment, we assessed the potential modulation of effectors of lipolysis/lipogenesis in adipose. In visceral adipose samples, we found no significant effects on the phosphorylation or expression of any of these markers, suggesting little effect of rapamycin. However, at the time of sacrifice, rapamycin-treated marmosets were not significantly leaner than control animals. Due to limitations of the design of this study, we could not determine whether these effectors were altered by rapamycin at earlier time points when fat mass was reduced by treatment (Fig. 1B).

## DISCUSSION

Prior to assessing the effectiveness of rapamycin as an anti-aging treatment in humans, it is first necessary to elucidate the potential effects on long term health outcomes. In particular, there has been a great deal of controversy and inconsistent results in the clinical studies of rapamycin, which have highlighted the potential increased risk for metabolic defects such as hyperlipidemia and hyperglycemia that are consistent with increasing risk of cardiovascular disease and type 2 diabetes [30]. However, the effects of this drug on relatively healthy humans are largely unknown. A recent short term study (6 weeks) of elderly patients given doses of a rapamycin analog found few side effects significant from placebo control subjects and reported a significant increase in serological response to flu vaccination, however, this study did not examine any markers of metabolic health in these subjects during treatment [31]. In order to assess potential consequences of long term rapamycin treatment on primate metabolic health we tested the effects of rapamycin on a group of healthy, aged, non-human primates, the common marmoset. We previously reported the ability to reliably and routinely dose socially housed marmoset monkeys with yogurt mixtures containing eudragit encapsulated rapamycin [29]. Dosing with 0.4 mg/day resulted in average blood rapamycin levels of 5.2 ng/mL, which is well within the range found in studies of other model species, and humans [1-5]. Further, we previously reported evidence of suppressed phospho-rpS6 in PBMC samples of rapamycin subjects suggesting down-regulation in mTORC1. In this study we reported several markers that suggest that chronic oral dosing with eudragit-encapsulated rapamycin has little impact on the metabolic status of marmosets.

Many have proposed that rapamycin is a mimic of calorie restriction which is the gold-standard for anti-aging intervention resulting in both extended life span and health span in many rodent models. Rapamycin has been shown to suppress mTOR activity, potentially decrease weight and fat mass, and extend healthspan and life span in a similar manner as calorie restriction [1-4]. In marmosets we demonstrated no overall change in body weight while being treated with rapamycin, but the marmosets did have significant loss of body fat. However, the loss of body fat stabilized at approximately five months of treatment and this time point was associated with an increase in food intake of rapamycin subjects. While we are unable to elucidate the underlying mechanisms for the sudden shift in dietary intake at 5 months of dosing, it is interesting to consider the possibility that the rapamycin-dosed animals altered caloric intake in response to the fat mass loss [32].

One of the major reported risks of rapamycin administration clinically is the development of new-onset type 2 diabetes as shown in clinical studies of kidney transplant patients treated with rapamycin analogs [33, 34]. However, the interpretation of these data is complicated by several factors including the impaired health status of the subjects and the use of combination therapies using additional drugs that are known to cause metabolic impairment on their own. Recent clinical studies of kidney transplant patients suggest that Tacrolimus rather than Sirolimus may be the leading cause of new onset diabetes within 10 weeks of treatment, however all research suggests that continued examination of monotherapies are needed to elucidate the side effects of each immunosuppressant [35, 36]. In rodents, the chronic administration of rapamycin as a mono-therapy has often been shown to impair glucose metabolism. For example, both inbred and genetically heterogeneous mouse strains develop glucose intolerance with oral administration of encapsulated rapamycin [11, 37, 38]. In inbred C57BL/6 mice, but not genetically heterogeneous mice, rapamycin is also associated with the development of insulin resistance. Interestingly, these effects of rapamycin on metabolism are dependent on both dose of rapamycin and sex of subjects [38] and do not appear to be permanent alterations as the metabolic defects can be reversed by ending rapamycin treatment [11]. Similarly, rapamycin treatment to normoglycemic, pre-diabetic *P. obesus* treated with rapamycin display heightened hyperglycemia and increased insulin resistance in part by reducing pancreatic β-cell function. In this rodent model it was suggested that rapamycin exacerbated the pre-existing diabetic symptoms and metabolic disorder in high risk animals [39]. Alternatively the effect of rapamycin is thought to mimic the metabolic changes associated with starvation diabetes or Type 0 diabetes [40, 41, 42]; which is often thought to be a positive, adaptive form of metabolic changes associated with enhanced metabolic efficiency and decreased risk of true diabetes. Evidence of enhanced insulin signaling following long term rapa [9], and intermittent rapa [11], as well as little evidence for detrimental mitochondrial function following rapamycin [42], supports the hypothesis that rapamycin may in fact be inducing changes similar to starvation diabetes [13, 40-42]. Interestingly, in our current study we found no evidence that rapamycin negatively impairs glucose metabolism in marmosets. One possible interpretation of these findings could be that rapamycin treatment might negatively and significantly affect only subjects that are predisposed to metabolic disease. This also might explain some of the slight differences in rapamycin's effect on glucose metabolic dysfunction in C57BL/6 but not genetically mixed mice. Another possible explanation could be the length of treatment utilized here. Recent studies have suggested a bi-phasic effect of rapamycin on glucose metabolism, with long-term administration of rapamycin to mice associated with increased, rather than reduced insulin sensitivity [8, 9, 44]. However, others have shown rapamycin in eudragit-encapsulated form (as we used in this study) does not show this bi-phasic effect, but rather continuously impairs glucose metabolism in mice [11,37]. In part, the metabolic impairments of rapamycin are thought to be due to inhibition of mTORC2 rather than mTORC1 [45]; while we previously showed that this rapamycin dose was sufficient to inhibit mTORC1 [29], it may not be sufficient to inhibit mTORC2 signaling in the marmoset and thus minimize the presumed metabolic defects of rapamycin treatment.

The increased risk of hyperglycemia with rapamycin treatment has been attributed to increased hepatic gluconeogenesis in rodent studies. In both mice and rats, rapamycin treatment significantly increases hepatic glucose production following injection with pyruvate and increases the expression of the gluconeogenic effectors like PCK1 and G6Pase [45, 46]. In this study, we also confirmed that a significant increase in PCK1 with rapamycin treatment, but paradoxically, found a significant decrease in G6Pase. While the rise in PCK1 would be consistent with increased gluconeogenesis, the decrease in G6Pase might be interpreted as inhibiting this process. It is not clear why rapamycin treatment has this contrasting effect in marmosets, but this might explain why marmosets did not display hyperglycemia with this treatment.

In addition to its well-known roles in cell survival and growth, recent studies have linked the mTOR signaling pathway with the regulation of lipid metabolism [47]. However, the direct effects of rapamycin on lipid metabolism have often been contradictory in published reports. For example, rapamycin has been reported to both improve and impair fatty acid oxidation in skeletal muscle cell lines [44, 48]. Reports regarding rapamycin's effects on lipid utilization in vivo in rodents are similarly inconsistent; rapamycin has been reported to decrease, increase or not effect fat accumulation among several different studies [1, 11, 37, 38, 49, 50]. Here, we found that rapamycin significantly reduced fat mass in marmosets at early time points in our treatment regime. However, after approximately 5 months of treatment, fat mass no longer differed between rapamycin-treated and control marmosets and we found no evidence for differences in lipolysis or lipogenesis in adipose tissue collected at the end of this study. Interestingly, this change coincided with a significant increase in food consumption among the rapamycin-treated marmosets. This alteration could represent a compensatory effect for the long-term inhibition of mTOR signaling. Further temporal studies regarding the effect of rapamycin in this model will be necessary to address this possibility.

Treatment with rapamycin as an intervention in the aging process for humans offers many possibilities but some studies have reported deleterious side effects that raise concern regarding the efficacy of this treatment. This study represents the first to examine the metabolic consequences of rapamycin dosing in healthy non-human primates. We have reported here evidence that long term rapamycin treatment at a dose that has been used in previous studies and reduces mTOR signaling in marmosets [29] does not result in notable negative side-effects on metabolic function in healthy marmosets. We believe that marmosets offer a unique non-human primate model that will allow detailed evaluation of the effect of potential anti-aging treatments on primate metabolic function, dietary intake, and activity patterning.

## MATERIALS AND METHODS

### Subjects

The subjects for this study were common marmosets (*Callithrix jacchus*) housed at the Southwest National Primate Research Center. Basic husbandry and housing for this colony have been described previously [51]. Thirteen subjects between the ages of 7.1 and 9.1 years were housed as female-vasectomized male pairs. Four pairs received daily oral dosing of 1.0 mg/kg/day (0.40 mg/day) rapamycin in a yogurt vehicle via syringe for 14 months as described [29]. Two pairs (5 subjects, one male died mid-way through the study and was replaced with another male) received daily doses of empty eudragit capsules in yogurt as control. Throughout the long term dosing regimen several markers of metabolic health were assessed.

### Body composition

Marmoset lean and fat mass was assessed monthly via quantitative magnetic resonance (QMR) imaging using an Echo MRI unit [26]. Unsedated animals were placed in a plastic tube which was then inserted into the magnetic chamber with scans taking less than 2 minutes on average for each animal. Animals were weighed biweekly throughout the project by placing a scale within the cage and rewarding the animal's for maintaining position on the scale.

### Caloric intake

Subjects participated in a 2 day food intake trial once per month for the length of the trial [28]. For these trials the subjects were separated from each other within the cage and fed their daily base diet consisting of two feed types from Harlan Teklad and Purina. Samples of each diet were taken from each prepared batch, frozen and stored until analysis. Diet fed to the subjects was weighed prior to feeding. After 24 hours all remaining food was removed and weighed, and fresh food was weighed and fed. After 48 hours all remaining food was removed and weighed and the subjects were returned to normal housing and feeding schedule. Samples were dried and dry weight consumption and caloric consumption was calculated and averaged over the 48 hour period.

### Activity

Daily activity patterns were assessed with the Mini actiwatch (CamNtech) which were placed in a marmoset pouch (Lomar) on a ferret harness (Petco). Subjects were gradually habituated to the ferret harnesses over the course of three weeks, increasing time in the harness incrementally throughout training until 24 hours in the harness had been achieved. The miniwatches are data loggers that batch data in 15 second epochs. For these trials animals were separated from each other within the cage and placed in harnesses with the actiwatch in the pouch secured across the back of the animal. Animals remained in the harness for 48 hours of data collection during which normal husbandry and feeding continued. At the end of the trial the animals were captured in transfer boxes and the harnesses were removed. Data was downloaded from the device. The activity counts from the first 15 minutes and last 15 minutes of the collection were removed from analysis as these represented handling and cage manipulation.

### Blood chemistry

Each month animals were fasted overnight and 2 ml of blood were drawn to assess circulating triglyceride concentrations, fasting glucose and insulin concentrations. Fasting glucose was determined immediately following the blood collection via glucometer. Blood was collected into serum separator tubes, spun and frozen in −80°C until further analysis. Triglyceride concentrations were assessed at the SNPRC clinical pathology lab. Samples were shipped to Wisconsin for analysis of insulin concentrations as described [52].

### Glucose challenge

Animals were fasted overnight prior to an oral glucose tolerance test [24], and placed in a restraint device used for blood collection to which they had previously been habituated. An EDTA coated needle and syringe were used to collect 0.5 ml of blood from the femoral vein for the baseline bleed. The animals were then dosed orally with a 40% dextrose solution receiving a calculated glucose dose equal to 0.5% of their current body weight. Subjects remained in the restraint for a 15 and 30 minute post dose blood sample drawn from the tail vein via an EDTA coated butterfly needle. Subjects were removed from the restraint device following the 30 minute sample and placed in a transport box until the 60 minute sample, and this was repeated for the 120 minute sample. The 15, 60 and 120 minute samples were glucometer reads only. For the 30 minute sample 0.5 ml of blood was collected for further analysis. Following the 120 minute bleed the animals were returned to their home cage and fed. The 0 and 30 minute samples were spun and frozen until shipment to Wisconsin for insulin assay analysis.

### Immunoblots

Total protein extracts were isolated from liver and visceral fat tissue that had been snapped frozen in liquid nitrogen after sacrifice and stored at −80°C until use. Protein extracts were homogenized in RIPA buffer with additional protease and phosphatase inhibitors (Thermo Scientific, Rockford, IL, USA), centrifuged at 14,000g at 4°C for 15 minutes, and then stored at −80°C until needed. Equal amounts of protein samples were separated electrophoretically by SDS-PAGE and then transferred to polyvinylidene difluoride membrane (Millipore, Billerica, MA, USA). Primary antibodies and their sources used in this study: adipose triglyceride lipase (ATGL), pyruvate carboxylase (PCB), glucose-6-phosphatase α (G6Pase), glucose-6-phosphate isomerase (GPI), and actin from Santa Cruz (Santa Cruz CA), peroxisome proliferator-activated receptor γ (PPARγ), phospho-pyruvate dehydrogenase kinase (p-PDK1), pyruvate dehydrogenase kinase (PDK1), phosphoenolpyruvate carboxykinase 1 (PCK1) from Cell Signaling (Beverly MA), sterol regulatory element-binding protein 1 (SREBP1) from Abcam (Cambridge MA) and deptor from Millipore with all alkaline phosphatase-conjugated secondary antibodies (anti-rabbit and anti-mouse) from Santa Cruz. Protein bands on immunoblots were detected using ECL reagent and analyzed using ImageJ.

### Analyses

Variables of interest included body mass, fat mass, fat-free mass, 24 hour total actimeter counts, 24 hour caloric intake, triglyceride concentration, fasting glucose concentration, fasting insulin concentration, and glucose AUC following an oral glucose challenge. For each variable, the change in pre- Rapa dose value to post- Rapa dose value, measured following (one month) of dosing was compared for control subjects versus rapamycin-treated subjects in a two-way, repeated measures ANOVA. An additional repeated measures ANOVA was used to compare values within each rapamycin-treated subject over time for the entire dosing period. Comparisons of tissue protein activity were done using MANOVA with Bonferroni corrections. Analyses were conducted using GraphPad/Prism and SPSS 13.0.
